# A “One-Stop” Screening Protocol for Haemoglobinopathy Traits and Iron Deficiency in Sri Lanka

**DOI:** 10.3389/fmolb.2019.00066

**Published:** 2019-08-09

**Authors:** Angela Allen, Shiromi Perera, Luxman Perera, Rexan Rodrigo, Sachith Mettananda, Agnes Matope, Ishari Silva, Nizri Hameed, Christopher A. Fisher, Nancy Olivieri, David J. Weatherall, Stephen Allen, Anuja Premawardhena

**Affiliations:** ^1^MRC Weatherall Institute of Molecular Medicine, John Radcliffe Hospital, University of Oxford, Oxford, United Kingdom; ^2^Department of Clinical Sciences, Liverpool School of Tropical Medicine, Liverpool, United Kingdom; ^3^Faculty of Medicine, University of Kelaniya, Colombo, Sri Lanka; ^4^Thalassemia Care Unit, North Colombo Teaching Hospital, Ragama, Sri Lanka; ^5^Tropical Clinical Trials Unit, Liverpool School of Tropical Medicine, Liverpool, United Kingdom; ^6^Pediatrics, Medicine, and Public Health Sciences, University of Toronto, Toronto, ON, Canada

**Keywords:** haemoglobinopathy traits, iron deficiency, osmotic fragility, dichlorophenolindophenol, red cell indices

## Abstract

**Introduction:** The high frequencies of carriers of severe haemoglobinopathies and of iron deficiency in Southeast Asia require reliable and affordable tests to improve on current screening procedures.

**Objectives:** We evaluate a “one stop” approach using the THALCON dichlorophenolindophenol (DCIP) and one-tube osmotic fragility (OF) tests and measurement of Zinc Protoporphyrin (ZPP) to detect and distinguish HbE and β-thalassaemia traits from iron deficiency. We compare findings with current screening practice in Sri Lanka that relies on the identification of low mean red cell volume and/or mean red cell hemoglobin for this purpose.

**Methods:** Between November 2017 and May 2018, we undertook a cross-sectional survey of secondary school students in Gampaha district, Sri Lanka. The THALCON–DCIP and OF tests were compared to capillary electrophoresis (CE), used as a gold standard to detect haemoglobinopathies. ZPP was measured in whole blood. Plasma ferritin and C-reactive protein (CRP) were measured in students with a raised ZPP concentration.

**Results:** We collected venous blood samples from 1,324/1,332 (99.4%) students. The median age of the students was 17 (IQR 16–18) years, all were Sinhalese and 814/1,324 (61.5%) were female. CE identified 3 students with HbE trait and 26 students with β-thalassaemia trait. The THALCON–DCIP test was positive only in the 3 students with HbE (sensitivity 100%, 95% CI 29.2–100.0; specificity 100%, 95% CI 99.7–100.0). The THALCON–OF test identified all 26 students with β-thalassaemia trait (sensitivity = 100%, 95% CI 86.8–100.0) and 287 students with a normal CE result (specificity = 77.9%; 95% CI 75.5–80.1). It was also positive in 2/3 (66.7%) students with HbE trait. Iron deficiency (ZPP > 70 μmol/mol heme) was present in 118/1,240 (9.5%) students with a normal hemoglobin genotype, all of whom had plasma ferritin <15 ng/ml and CRP <5 mg/L.

**Conclusion:** This one–stop approach offers reliable and affordable population screening for both haemoglobinopathy traits and iron deficiency in resource-limited settings where these conditions are common and ensures that iron supplements are targeted only to those who require them. Further work is warranted to refine the OF test to reduce the number of false positive results.

## Introduction

It is estimated that >7% of the world's population carry a hemoglobin variant, resulting in 300,000–500,000 babies born each year with a significant hemoglobin disorder. Ninety percent of these births occur in low or middle-income countries (Weatherall, 2010). Hemoglobin disorders are estimated to account for at least 3.4% of under-five deaths (Modell and Darlison, 2008).

A recent survey of 23 thalassaemia treatment centers in Sri Lanka identified 1,219 patients with β-thalassaemia major, 360 with HbE/β-thalassaemia, and 50 with sickle β-thalassemia (A Premawardhena, personal communication). Patients with β-thalassaemia major require life-long intensive clinical management and monthly blood transfusion (now referred to as transfusion-dependent thalassaemia TDT). The clinical course of HbE/β-thalassaemia is more variable, ranging from mild (non-transfusion dependent thalassaemia NTDT) to severe anemia. In both conditions, in the absence of early and effective iron-chelation therapy, transfusional iron overload may result in liver and cardiac dysfunction, and endocrine abnormalities including growth disturbances and glucose intolerance. Often, patients experience a severely reduced quality of life, suffer from social stigmatization, and may have difficulties in securing employment. In higher-resource settings, survival now extends to the fifth or sixth decade, but this is not true in lower income settings where the costs and complexities of care mean that survival is curtailed (Cunningham et al., 2004). The cost of care for thalassaemia in Sri Lanka is estimated to be more than 5% of the national health budget each year (de Silva et al., 2000). In a survey of 7,526 school children in Sri Lanka, the frequency of β-thalassaemia and HbE traits varied from 0.0 to 8.1% and 0.0 to 1.9%, respectively, according to district (Premawardhena et al., 2017).

Iron deficiency is the most common micronutrient disorder and causes anemia in up to 30% of individuals throughout the world. In adolescents, anemia impairs school performance and resistance to infection (WHO/UNICEF/UNU Iron deficiency anaemia: Assessment, Prevention and Control, 2001; Black, 2003; Zimmerman and Hurrell, 2007). In the above survey of Sri Lankan school children and adolescents, the frequency of iron deficiency was 19.2% (Allen et al., 2017).

A national haemoglobinopathy screening programme began in Sri Lanka in 2005 with the goal of reducing births of severely affected children. The screening programme targets school children, adolescents and young people, to allow individuals to make informed choices regarding future marriage partners and child-bearing. In view of the high cost of the definitive tests for haemoglobinopathy detection (Capillary Electrophoresis (CE), high-performance liquid chromatography and DNA analysis), in Sri Lanka, as in many Asian countries, population screening relies on detecting low mean red cell volume and/or mean red cell hemoglobin (hereafter “low red blood cell indices”) to identify possible carriers of haemoglobinopathies who require further investigation. However, this approach has several limitations. Reliable measurement of red cell indices requires automated hematology analysers that are maintained and calibrated regularly, with quality controls run daily. Accuracy may vary according to the instrument used. Low red cell indices also occur in iron deficiency, complicating differentiation from haemoglobinopathy traits. Finally, red cell indices may be normal in carriers of unusually mild β-thalassaemia alleles and in HbE trait (Weatherall, 2001; Fucharoen et al., 2004; Sanchaisuriya et al., 2005; Galanelo, 2012; Singha et al., 2019) and in people who inherit more than one haemoglobinopathy trait (e.g., α-thalassaemia together with β-thalassaemia (Penman et al., 2015).

The DCIP and OF tests have been proposed as simple visual screening tests for the detection of carriers of HbE and β-thalassaemia, respectively (Sanchaisuriya et al., 2005). The DCIP test, first described in 1976 (Kulapongs et al., 1976), is based on the principle that HbE is an unstable hemoglobin that when added to the blue dye dichlorophenolindophenol, at neutral pH, is oxidized, forming a precipitate (Kulapongs et al., 1976; Old, 2012). In the OF test, thalassaemic red blood cells are more resistant to haemolysis than normal red blood cells because of their small size and low cellular hemoglobin content, and when added to hypotonic buffered saline, typically of 0.36% (w/v) concentration, produce a turbid solution (Parpart et al., 1947; Winichagoon et al., 2002; Old, 2012; Ansari et al., 2014). Both the DCIP and OF tests have been successfully used as screening tests in Thailand since 1990, and reagents available in kit form have been developed and extensively validated. In previous studies, the sensitivity and specificity of the tests when used in combination for the detection of HbE and β-thalassaemia traits ranged between 99.2 and 100% and 79.3–97.1%, respectively (Sangkitporn et al., 2005; Savongsy et al., 2008; Viprakasit and Ekwattanakit, 2018).

We have evaluated the THALCON-DCIP and THALCON-OF tests in 60 parents of patients with either β thalassaemia or HbE/β- thalassaemia attending the Thalassemia Care Unit, North Colombo Teaching Hospital, Ragama, Sri Lanka. The DCIP test identified all 15 parents with HbE-trait. The OF test correctly identified all 45 parents with β-thalassaemia trait and was also positive in 5/15 HbE traits. There were no false negatives in either test.

The ZPP test is based on the principle that during the final stage of heme synthesis in the bone marrow, if the iron supply is limited or unavailable, zinc, instead of iron, is incorporated into the protoporphyrin ring of the hemoglobin molecule and accumulates in red blood cells (WHO, 2007). The World Health Organization recognizes the usefulness of ZPP as a screening test for iron deficiency in population surveys in low–resource settings (WHO, 2007), and ZPP was used to identify iron deficiency in one of the largest randomized control studies of iron supplements in children in Zanzibar (Sazawal et al., 2006).

We took the opportunity to join the national haemoglobinopathy screening program during its annual survey of secondary schools in Gampaha district, Sri Lanka to evaluate the THALCON-DCIP and THALCON–OF test kits and the measurement of ZPP as a “one-stop” screening procedure for the detection of haemoglobinopathy traits and iron deficiency. We compared this approach to the measurement of low red cell indices, the test currently used by the national screening program.

## Methods

### School Surveys

Between November 2017 and May 2018 we recruited students attending 9/172 secondary schools with Advanced Level classes from Gampaha district, Sri Lanka. Schools were purposefully selected so that they were geographically spaced within the Gampaha district. Study personnel visited each school to explain the purpose of the study to students and teachers. All students in grades 10–13 (>14 years of age) were invited to partake. Signed, informed consent was obtained from parents/guardians; all students also provided verbal consent. Any student who reported feeling un-well on the day of the survey was not enrolled into the study. Approximately 150 students, drawn from across the four school years were enrolled from each school. Age, sex, ethnicity, and place of residence were recorded. In students with mixed ethnicity, that of the father was recorded.

### Laboratory Procedures

**Figure 1:**
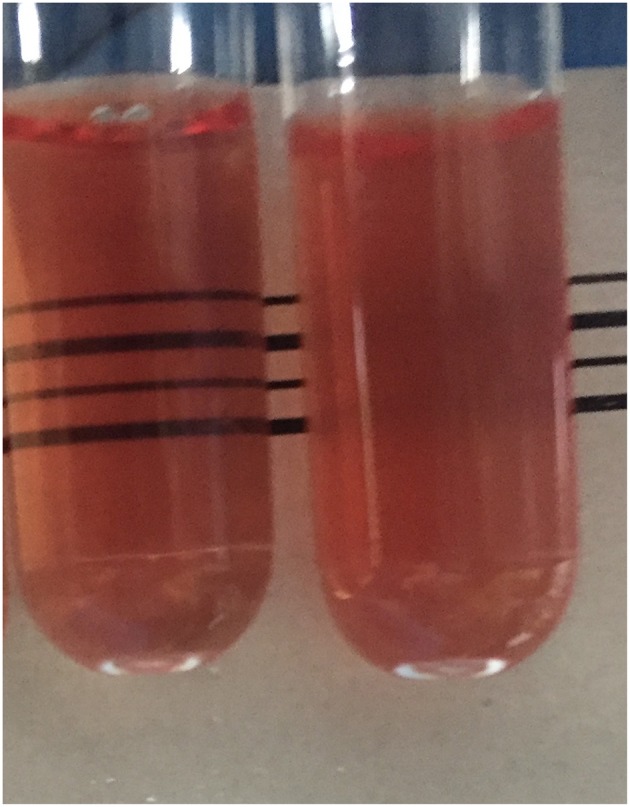
The clear pink solution observed in the tube on the left indicates a normal test result, whereas the turbid pink appearance of the tube on the right indicates a positive test result.

A 2.5 ml venous blood sample was collected into EDTA anticoagulant from each student. Samples were stored in a cool box and transferred to the laboratory within 3 h of collection for measurement of hemoglobin concentration, red cell indices (Beckman Coulter Ac.T diff analyser, Luton, UK), detection of hemoglobin variants by CE (Capillarys 2 Flex Piercing Instrument, Sebia, Lisses, France), and the THALCON- DCIP and THALCON-OF tests (Surathin International Company Ltd., Thailand), in accordance with the manufacturer's guidelines. Briefly, for the DCIP test, 20 μl whole blood was added to 2 ml DCIP solution, gently mixed by inversion and incubated at 37°C for 15 min. 20 μl of clearing solution was then added to each reaction and the results interpreted immediately. For the THALCON-OF test, 20 μl whole blood was added to 2 ml of a 0.36% buffered saline solution, gently mixed by inversion and incubated at room temperature for 5 min before the results were read. In both tests, a turbid pink solution indicated a positive result and a clear pink solution indicated a negative/normal result (see Figure 1). Positive and negative control blood samples were included for each set of tests.

ZPP was measured in whole blood using a front-faced haematofluorimeter and Protofluor reagent system (Helena BioSciences, South Shields, UK). The remaining samples were centrifuged, the buffy coats and plasma removed and stored at −20°C for further analyses. Plasma CRP and ferritin were measured by Enzyme Linked Immunosorbent assay (DCRP00, Biotechne, UK and DB59111, Tecan, UK, respectively) in samples with a raised ZPP to confirm iron deficiency and to explore whether inflammation may have resulted in a raised ZPP result.

To investigate possible causes of a positive OF test in samples with a normal CE result, DNA was extracted from buffy coat samples using QIAGEN DNA mini-kit (QIAGEN, UK) and α- globin genotype determined by Multiplex polymerase chain reaction (Tan et al., 2001; Shaji et al., 2003).

### Statistical Methods

Categorical variables were summarized using counts and percentages. Continuous variables tended to have skewed distributions; they were described using median and interquartile range and compared using the Mann-Whitney *U* test. Sensitivity and specificity and the corresponding 95% confidence intervals (CI) of the DCIP and OF tests were calculated with capillary electrophoresis (CE) as the gold standard. Considering the small number of positive results for the DCIP test, the exact 95% CIs were obtained. All the estimates whose corresponding 95% CI did not span 50% (i.e., proportion = 0.5) were considered significant and all tests were performed at 5% significance level. All data analysis was performed using SPSS statistical software version 25.

### Ethical Approval

The study and the consent procedures were approved by the Ethics Committee, University of Kelaniya, Sri Lanka and Oxford University Tropical Research Ethics Committee, Oxford, UK. The study was conducted in accordance with the declaration of World Medical Association (2008).

## Results

A total of 1,332 students were enrolled and venous blood samples were collected from 1,324 (99.4%). The 8 students for whom it was not possible to obtain a blood sample have been excluded from the analysis. The median age of the students was 17 (IQR 16–18) years; all were Sinhalese and 814/1,324 (61.5%) were female.

The sensitivity and specificity for the screening tests are shown in Table 1. Laboratory findings according to hemoglobin genotype and sex are shown in Supplementary Table S1.

**Table 1:** Sensitivity and specificity of screening tests for haemoglobinopathy traits compared to capillary electrophoresis as the gold standard.

**Test**		**Capillary electrophoresis**		**Sensitivity** **(95% CI)**	**Specificity** **(95% CI)**
		**Pos**	**Neg**		
Capillary electrophoresis					
HbE trait		3	1,321		
β-thalassaemia trait		26	1,298	-	-
HbE trait or β-thalassaemia trait		29	1,295		
Thalcon DCIP test (HbE)	**Pos**	3	0	100.0	100.0
	**Neg**	0	1,321	(29.2–100.0)	(99.7–100.0)
Thalcon OF test (β-thalassaemia trait)^*^	**Pos**	26	287	100.0	77.9
	**Neg**	0	1,009	(86.8–100.0)	(75.5–80.1)
Thalcon DCIP and Thalcon OF	**Pos**	29	287	100.0	77.8
	**Neg**	0	1,008	(88.1–100.0)	(75.5–80.1)
Low red cell indices (MCV <80 fl or MCH <27 pg)	**Pos**	29	409	100.0	68.4
	**Neg**	0	884	(88.1–100.0)	(65.8–70.9)

* *The two students with HbE trait and positive OF test are excluded*.

### Haemoglobinopathy Traits

CE identified hemoglobin variants in 29 (2.2%) students. Three students were HbE trait and the DCIP test was positive only in these 3 samples (sensitivity = 100%, 95% confidence interval (CI) 29.2–100.0; specificity = 100%, 95% CI 99.72–100.0). Twenty-six students had raised HbA_2_, indicative of β-thalassaemia trait. The OF test correctly identified all 26 students with β-thalassaemia trait but was also positive in 287 samples with normal CE results (sensitivity = 100%, 95% CI 86.8–100.0; specificity = 77.9%, 95% CI 75.5–80.1). The OF test was also positive in 2 of 3 students with HbE trait. Overall, in the detection of either β-thalassaemia trait or HbE trait, the combination of the DCIP and OF tests had a sensitivity of 100% (95% CI 88.1–100.0) and specificity of 77.8% (95% CI 75.5–80.1).

### Low Red Cell Indices

Low red cell indices (mean cell volume (MCV) <80 fl or mean cell hemoglobin (MCH) < 27 pg) were present in all 26 students with β-thalassaemia trait and the 3 students with HbE trait but were also present in 409/1,293 (31.6%) students with a normal CE result (sensitivity = 100%; 95% CI 88.1–100.0; specificity = 68.4%, 95% CI 65.8–70.9).

### Iron Deficiency

ZPP was raised (>70 μmol/mol heme) in 141/1,314 (10.7%) students including 14/26 (53.8%) with β-thalassaemia trait and 9 students with α-thalassaemia trait. In 118/1,240 (9.5%) students with a normal hemoglobin genotype and raised ZPP, all had normal plasma CRP (<5 mg/L) and low plasma ferritin concentrations (<15 ng/ml), confirming iron deficiency.

In the 14 students with β-thalassaemia trait and raised ZPP, plasma CRP was normal in 12/14 (85.7%) and ferritin in 13/14 (92.9%); only one student had iron deficiency.

Sufficient plasma was available to measure CRP and ferritin in 8/9 students with α-thalassaemia trait and a raised ZPP. Iron deficiency was confirmed 7/8 (87.5%) students; one student had a normal plasma ferritin and a raised CRP.

## Analysis of Unexplained-Positive of Tests

### α-globin Genotype

Amongst the 287 students with an unexplained positive OF result, sufficient DNA was available to determine α-globin genotype in 286/287 (99.7%). Forty-five students had α-thalassaemia; 35 with -α^3.7^/ αα, 4 with -α^3.7^ /-α^3.7^ and 6 with –α^4.2^/ αα. The less common α-globin gene mutations – –^SEA^, – –^FIL^, – –^MED^, – –^THAI^, – α^−20.5^ were not present in any sample.

### Low Red Cell Indices and Anemia

**Figure 2:**
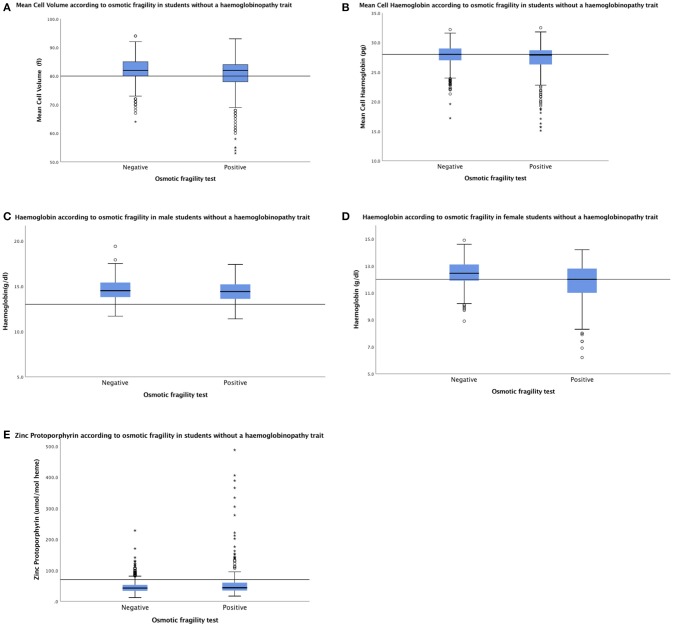
Median (IQR) MCV **(A)** and MCH **(B)** were similar in 235 students with an unexplained positive OF result and 1,005 students with a true negative OF result: 82.0 fl (77.0–84.0) and 27.8 pg (25.7–28.6) vs. 82.0fl (80.0–85.0) and 28.0 (26.9–29.0), respectively. Median (IQR) Hb was 14.4 g/dl (13.6–15.2) and 12.0 g/dl (10.9–12.8) in 96 males **(C)** and 139 females **(D)**, respectively, with an unexplained positive OF result vs. 14.5 g/dl (13.8–15.4) and 12.5 g/dl (11.9–13.1) in 383 males and 622 females with a true negative OF result. Median (IQR) ZPP was similar in 235 students with an unexplained positive OF result and 1,005 with a true negative OF result; 44 μmol/mol heme (add IQR) and 43 μmol/mol heme (add IQR), respectively **(E)**. Horizontal lines inside the box show the median value, box length is the interquartile range and whiskers show the range, excluding outliers. Outlying values 1.5–3, or > 3 box lengths from the upper and lower edge of the box are shown as open circles and stars, respectively. Reference lines shows the lower limit of the normal range for MCV, MCH, and Hb and the upper limit of the normal range for ZPP.

Mean cell volume, mean cell hemoglobin and hemoglobin concentration were measured in 235/242 (97.1%) of the remaining students with an unexplained positive OF test, and median values were similar to those of 1,005 students with a true negative OF result (Figures 2A–D). However, the frequency of low red cell indices and of anemia in the two groups were significantly different (85/235 (36.2%) and 280/1,005 (27.9%; *p* = 0.0137), respectively for low red cell indices and 75/235 (31.9%) and 202/1005 (20.1%; *p* = 0.0002) for anemia.

### Iron Deficiency

Median ZPP was similar in 235 students with an unexplained positive OF result and 1,005 with a true negative OF result; 44 μmol/mol heme and 43 μmol/mol heme, respectively (Figure 2E). However, the frequency of raised ZPP was greater in students with an unexplained positive OF test (47/235; 20.0%) than those with a true negative result (77/1,005; 7.7%; *p* < 0.0001).

## Discussion

The THALCON-DCIP and THALCON-OF tests performed well in our hands, were quick and easy to use and required minimal laboratory equipment. The inclusion of a clearing agent in the DCIP kit, used to decolorize the blue DCIP dye at the end of the incubation period, allowed the tests to be read easily.

Although the THALCON–DCIP test had 100% sensitivity and specificity for carriers of HbE, the low number of cases in our study resulted in a wide 95% confidence interval for sensitivity (29.2–100.0). Low red cell indices, the current screening criteria used in Sri Lanka, also identified all HbE carriers in this study. However, low red cell indices are not always a feature of HbE trait, and we have reported previously that in a larger survey of 7,526 secondary school students in Sri Lanka, red cell indices were normal in 3/38 (7.9%) of students with HbE trait (Premawardhena et al., 2017). Similarly, in a study of pregnant women in Thailand, 14/93 (15.5%) HbE traits had normal red cell indices (Sanchaisuriya et al., 2005). Further evaluation of the THALCON–DCIP test in populations with a low frequency of HbE carriage will require large numbers. For example, In Sri Lanka where the overall prevalence of HbE trait is 0.23%, with a lower 95% confidence limit for sensitivity to be >90% and 0.80 probability, a sample size of 18,261 participants would be required and 53,479 participants would be required for the lower 95% confidence limit for sensitivity to be >95%.

The THALCON-OF test identified all carriers of β-thalassaemia, and the greater prevalence of this haemoglobinopathy in Sri Lanka allowed a more reliable assessment of sensitivity (95% CI 86.8–100.0). Use of low red cell indices had a similar sensitivity. Although the THALCON-OF test identified many false positives (specificity 77.9%; 95% CI 75.5–80.1), specificity was better than when using low red cell indices (specificity = 68.4%; 95% CI 65.8–70.9). Using low red cell indices would have resulted in 438/1,322 (33.1%) samples requiring expensive confirmatory tests compared to 315/1,324 (23.7%) with the OF test (representing approximately 30% fewer tests).

Although ZPP can be raised in conditions other than iron deficiency, such as inflammation and some haemoglobinopathies (Thomas et al., 1977; Labbé, 1992; WHO, 2007; Parischa and Drakesmith, 2016), we found that the inclusion of ZPP measurement in this study was helpful in identifying students who were truly iron deficient as confirmed by reduced plasma ferritin and normal CRP concentrations, excluding inflammation. However, we did not measure CRP and ferritin in students with a normal ZPP and it is possible that some students with earlier stages of iron deficiency may have been missed. Raised ZPP is also useful in idenitfying people with lead poisoning (Thomas et al., 1977; Labbé, 1992; WHO, 2007; Parischa and Drakesmith, 2016); however, this is unlikely to be common in Sri Lanka following the introduction of unleaded petroleum in 2002 (Senanayake et al., 2004).

Almost one third of this study population had low red cell indices. Because low indices are a feature of both iron deficiency and some haemoglobinopathy traits, current screening practice in Sri Lanka means that staff are often faced with a dilemma when deciding on further investigations to identify the cause of the low indices. In addition, we have reported that low red cell indices not attributable to either iron deficiency or haemoglobinopathy traits are also common in this population (Allen et al., 2017; Rodrigo et al., 2018). Because the definitive tests for haemoglobinopathy traits are expensive and iron deficiency is more common in Sri Lanka, screening staff recommend that individuals with low indices repeat their full blood count after a 3–6 month course of oral iron, to exclude iron deficiency as the cause. If at follow-up the red cell indices remain low, the definitive test is then performed. Based on these current screening procedures, in this study, iron supplements would have been recommended to 438 (33.1%) students with low red cell indices. However, by measuring ZPP in combination with the DCIP and OF tests in a one-stop procedure this number would be reduced to 77/1,005 (7.7%) students, representing a considerable saving. Moreover, the risk of loss to follow-up that occurs with current screening practice would be limited. Avoiding the unnecessary use of iron supplements is important given that tolerance of oral iron is poor and iron increases the risk of infection (Murray et al., 1978; Drakesmith and Prentice, 2012; Kortman et al., 2012). Furthermore, the high frequency of the H63D variant of the haemochromatosis gene and the presence of haemoglobinopathy traits in Sri Lanka (Premawardhena et al., 2017; Allen et al., 2019) mean that it is important to ensure that iron supplements are targeted to only those who require them, to avoid the possible deleterious effects of increased iron availability in iron replete individuals.

Although our numbers were small, ZPP appeared to be a reliable marker of iron deficiency in α-thalassaemia but not in β-thalassaemia traits. Our findings in β-thalassaemia trait are concordant with other studies but are discordant with studies for α-thalassaemia (Tillyer and Tillyer, 1994; Graham et al., 1996). Additonal confirmatory tests for iron deficiency are required for students with haemoglobinopathy traits before recommending iron supplements.

Consistent with other studies of OF (Kattamis et al., 1981; Jopang et al., 2009), we observed a significant number of false positive results with the THALCON-OF test. DNA analysis confirmed that α-thalassaemia accounted for the positive OF result in about 20%, and low red cell indices, anemia or iron deficiency may also have contributed. It is possible that the concentration of saline used in the test (0.36%) was too high for the complete lysis of normal red blood cells, and using a weaker saline solution may have reduced the number of false positives. Supporting this hypothesis is a study of haemoglobinopathy traits in a rural population of Thai Khmer, in which a buffered saline solution of 0.34% reduced OF false positivity without compromising the sensitivity of the test (Fucharoen et al., 2004).

We consider that the combination of the OF, DCIP, and ZPP assays is a useful approach to screening for haemoglobinopathy traits and iron deficiency in population surveys in Sri Lanka, and has several advantages over the use of low red cell indices alone. These findings are directly relevant to populations in other low and middle-income countries in which haemoglobinopathies and iron deficiency are common. The study was conducted under research conditions by trained laboratory staff, and now needs to repeated in routine and remote settings, where laboratory expertise may be variable.

Regardless of the screening methods used, there is a pressing need to establish external quality assessment (EQA) programmes to monitor how well the tests are performed and interpreted by local staff, to ensure the accuracy and success of all haemoglobinopathy screening programmes. Indeed, an EQA programme implemented in Thailand has been shown to improve the performance of many screening laboratories (Prommetta et al., 2017).

## Conclusion

A combination of OF, DCIP, and ZPP assays improves upon current screening procedures for haemoglobinopathy traits that are currently based on identification of low red cell indices, and also identifies iron deficiency in a one-stop procedure. Further work should be undertaken to refine the OF assay in an effort to reduce the number of false positives. Further research should evaluate the performance and cost effectiveness of this one-stop approach in routine and remote laboratory settings.

## Data Availability

The datasets generated for this study are available on request to the corresponding author.

## Author Contributions

AA, DW, NO, SA, and AP designed the study. AA, SP, LP, RR, and CF performed the laboratory work. SP, LP, SM, IS, and NH performed the field work. SP performed the data entry. AA, AM, and SA performed the statistical analysis. AA and SA wrote the manuscript and all authors reviewed and approved the final draft.

### Conflict of Interest Statement

The authors declare that the research was conducted in the absence of any commercial or financial relationships that could be construed as a potential conflict of interest.
