# Supplementary material for: A “One-Stop” Screening Protocol for Haemoglobinopathy Traits and Iron Deficiency in Sri Lanka
Source: Front Mol Biosci. 2019 Aug 9;6:66. doi: 10.3389/fmolb.2019.00066 (PMC6696778; doi:10.3389/fmolb.2019.00066)
Supplement: Supplementary file 1 [file Table_1.DOCX]

**Supplementary table S1. Haemoglobin, osmotic fragility, DCIP, red cell indices and zinc protoporphyrin according to sex and haemoglobin genotype.**

|  | **Normal** | | **β –thalassaemia trait** | | **HbE-trait**^1^ | | **α-thalassaemia trait** | |
| --- | --- | --- | --- | --- | --- | --- | --- | --- |
|  | Male  n=480 | Female  n=770 | Male  n=13 | Female  n=13 | Male  n=2 | Female  n=1 | Male  n=21 | Female  n=24 |
| HbA (%)  median (IQR) | 97.3  (97.1-97.4) | 97.4  (97.3-97.6) | 94.2  (93.4-94.7) | 94.3  (93.7-94.8) | 70.3, 71.1 | 70.9 | 97.4  (97.3-97.5) | 97.6  (97.4-97.8) |
| HbA2 (%)  median (IQR) | 2.7  (2.5-2.8) | 2.5  (2.4-2.7) | 5.6  (5.3-5.9) | 5.1  (4.9-5.5) | 3.0, 3.9 | 3.7 | 2.5  (2.4-2.7) | 2.4  (2.1-2.5) |
| Hb(E%)  median (IQR) | - | - | - | - | 25.0, 25.4 | 25.4 | - | - |
| Thalcon Osmotic fragility test positive  n pos/n  (%) | 97/480  (20.2%) | 146/770  (19.0%) | 13/13 (100%) | 13/13 (100%) | 1/2  (50%) | 1/1 (100%) | 21/21  (100%) | 24/24  (100%) |
| Thalcon DCIP test positive  n (%) | 0/480  (0%) | 0/770  (0%) | 0/13  (0%) | 0/13  (0%) | 2/2  (100%) | 1/1  (100%) | 0/21  (0%) | 0/24  (0%) |
| ZPP (umol/mole heme)  median (IQR) | 28.3  (27.4-29.1)^2^ | 49.0  (39.0-62.0) ^3^ | 71  (46-97) | 74  (65-108) ^4^ | 38.0, 34.0 | 40.0 | 32.0  (27.0-43.0) | 50.5  (40.8-110.3) |
| Hb (g/dl)  median (IQR) | 14.5  (13.7-15.3) | 12.4  (11.8-13.0) ^5^ | 12.2  (11.9-13.1) | 11.0  (10.5-11.2) | 12.3, 13.2 | 12.6 | 14.1  (13.4-15.0) | 11.5  (10.5-12.6) |
| Low red cell indices  n pos/n (%) | 106/480  (22.1%) | 266/768  (34.6%) | 13/13  (100%) | 13/13  (100%) | 2/2  (100%) | 1/1  (100%) | 15/21  (71.4%) | 22/24  (91.7%) |
| MCV (fl)  median (IQR) | 83.0  (80.0-85.0) | 82.0  (79.0-85.0) ^5^ | 60.0  (58.8-63.0) | 59.0  (57.5-61.5) | 74.0, 77.0 | 74.0 | 79.0  (76.0-80.5) | 71.5  (65.8-77.5) |
| MCH (pg)  median (IQR) | 28.3  (27.4-29.1) | 27.7  (26.4-28.8) ^5^ | 19.3  (18.5-20.0) | 18.7  (17.3-19.3) | 24.4, 25.6 | 24.1 | 26.7  (25.3-27.4) | 23.8  (21.3-26.1) |

1. For the 3 students with HbE trait, individual values are shown instead of median (IQR)

Data missing from: ^2^3 subjects, ^3^9 subjects, ^4^1 subject and ^5^2 subjects.
